# Commentary: MicroRNA-221/222 Family Counteracts Myocardial Fibrosis in Pressure Overload-Induced Heart Failure

**DOI:** 10.3389/fcvm.2018.00095

**Published:** 2018-07-16

**Authors:** Boris Schmitz, Stefan-Martin Brand

**Affiliations:** Institute of Sports Medicine, Molecular Genetics of Cardiovascular Disease, University Hospital Muenster, Muenster, Germany

**Keywords:** heart failure, remodeling, micoRNAs, cardiac hypertrophy, exercise

Recently Verjans et al. (1) investigated the role of microRNA (miRNA)-221 and -222 in heart failure and cardiac remodeling. Using endomyocardial biopsies of patients with dilated cardiomyopathy (DCM) the authors found significantly reduced levels of miRNA-221-3p and -222-3p in patients with severe fibrosis compared to those with non-severe fibrosis. Subsequent investigations in mice suggested that inhibition of miRNA-221 and -222 aggravated pressure overload–induced heart failure potentially by targeting components of the transforming growth factor-beta (TGF-beta) pathway. The authors concluded that delivery of synthetic miRNA-221/-222 might be a future treatment option in heart failure (1). Since these results are partly in contradiction with earlier reports suggesting that elevated levels of miRNA-221 and -222 may actually cause heart failure, it might be worthwhile to look at the history of miRNA-221/-222 research in heart failure.

More than a decade ago, Sayed et al. (2) performed an early miRNA microarray screening of RNA extracted from mice hearts in which cardiac hypertrophy had been induced by transverse aortic constriction. At that time, the applied Atactic μParaflo microfluidics chip covered 334 mature miRNA probes (2), whereas today, with the latest miRBase version 22 release (www.mirbase.org/, GRCm38, March 2018) (3) 2,013 mature mouse miRNAs can be screened. The group detected miRNA-221 and -222 (among others) significantly upregulated 7–14 days post-intervention in hyperthrophic mice compared to control (2). Shortly after, these results were confirmed in an independent microarray approach by another group who induced pressure overload in mice by thoracic aortic banding (4). Based on these findings, Wang et al. (5) started to analyse miRNA-221 in cardiac hypertrophy including the generation of transgenic mice for the constitutive cardiac-specific overexpression of miRNA-221 (6) and miRNA-222 (7). Their studies suggested that (separate) overexpression of miRNA-222 and miRNA-221 caused cardiac dysfunction and heart failure leading to the conclusion that miRNA-221 and -222 are potential therapeutic targets for heart failure (6, 7), indicating that targeted reduction of the miRNAs would be favorable.

Additional evidence, especially for the role of miR-222 in cardiac hypertrophy and heart failure, came from an independent array-based screening (TaqMan rodent miRNAarray, A+B set v3.0, 641 mmu-miRNAs) of cardiac miRNAs from mice subjected to a ramp swimming exercise or voluntary wheel running model (8). The authors identified 16 regulated miRNAs (validated over both models) involved in physiological cardiac growth including miR-222 but not miR-221. Furthermore, suppression of miRNA-222 during exercise by LNA-anti-miRNA-222 injection prevented cardiomyocyte growth and inhibited cardiac hypertrophy and the overall increase in heart size. Treatment of sedentary mice with anti-miRNA-222 for 3 weeks did not affect heart weight (8). Subsequently, the group generated inducible cardiomyocyte-specific miRNA-222 transgenic mice in which they induced miRNA-222 overexpression at week 10–12 for 4 weeks. This led to a ~6.5-fold increase of cardiac miRNA-222 but did not affect heart size and cardiac function despite significantly higher miRNA-222 levels compared to exercise-induced miRNA-222 levels (2.8-fold in running model). The authors concluded that miRNA-222 is necessary for cardiac hypertrophy in exercise but is not sufficient to cause the exercised-heart phenotype (8). However, both groups presented data suggesting that miRNA-222 acts partly through the deactivation of cyclin-dependent kinase inhibitor p27(KIP1) (7, 8) which induces cell cycle arrest (9).

Taken together, it seems likely that miRNA-222 may indeed have cardioprotective effects in adult animals (and potentially humans) and in certain disease conditions such as heart failure rather than being detrimental. Several studies have shown that miRNA-222 may be specifically induced by physical exercise not only in athletes (10–12) but also in moderately trained healthy individuals (13) as well as patients with heart failure (8). It may therefore be of interest to initiate further studies to investigate to what extend miRNA-222 might be a useful marker to monitor cardioprotective exercise in primary and secondary prevention and rehabilitation of heart failure patients.

## Author contributions

BS has fully reviewed the original article, drafted the commentary, reviewed, and approved the final manuscript. S-MB has also reviewed the original article, assisted in drafting the commentary, reviewed and approved the final manuscript.

## Conflict of interest statement

The authors declare that the research was conducted in the absence of any commercial or financial relationships that could be construed as a potential conflict of interest.
